# Activation of Peripheral Blood Mononuclear Cells and Leptin Secretion: New Potential Role of Interleukin-2 and High Mobility Group Box (HMGB)1

**DOI:** 10.3390/ijms22157988

**Published:** 2021-07-26

**Authors:** Andrea Coppola, Barbara Capuani, Francesca Pacifici, Donatella Pastore, Roberto Arriga, Alfonso Bellia, Aikaterini Andreadi, Nicola Di Daniele, Renato Lauro, David Della-Morte, Giuseppe Sconocchia, Davide Lauro

**Affiliations:** 1 Department of Systems Medicine, University of Rome “Tor Vergata”, 00133 Rome, Italy; coppola@med.uniroma2.it (A.C.); barbara.capuani@uniroma2.it (B.C.); francesca.pacifici@uniroma2.it (F.P.); d.pastore3@inwind.it (D.P.); roberto.arriga@uniroma2.it (R.A.); bellia@med.uniroma2.it (A.B.); katia.andreadi@gmail.com (A.A.); didaniele@med.uniroma2.it (N.D.D.); lauro@uniroma2.it (R.L.); david.dellamorte@uniroma2.it (D.D.-M.); 2 Department of Medical Sciences, Fondazione Policlinico Tor Vergata, 00133 Rome, Italy; 3 Department of Human Sciences and Quality of Life Promotion, San Raffaele Rome Open University, 00166 Rome, Italy; 4 Institute of Translational Pharmacology, National Research Council Rome, 00133 Rome, Italy; giuseppe.sconocchia@cnr.it

**Keywords:** type 2 diabetes mellitus, toll-like receptors system, HMGB1, leptin

## Abstract

Activation of innate immunity and low-grade inflammation contributes to hyperglycemia and an onset of Type 2 Diabetes Mellitus (T2DM). Interleukin-2 (IL-2), leptin, High Mobility Group Box-1 (HMGB-1), and increased glucose concentrations are mediators of these processes also by modulating peripheral blood mononuclear cells (PBMCs) response. The aim of this study was to investigate if HMGB-1 and IL-2 turn on PBMCs and their leptin secretion. In isolated human PBMCs and their subpopulations from healthy individuals and naïve T2DM patients, leptin release, pro-inflammatory response and Toll-like Receptors (TLRs) activation was measured. After treatment with IL-2 and HMGB1, NK (Natural Killer) have the highest amount of leptin secretion, whilst NK-T have the maximal release in basal conditions. TLR4 (TAK242) and/or TLR2 (TLR2-IgA) inhibitors decreased leptin secretion after IL-2 and HMGB1 treatment. A further non-significant increase in leptin secretion was reported in PBMCs of naive T2DM patients in response to IL-2 and HMGB-1 stimulation. Finally, hyperglycemia or hyperinsulinemia might stimulate leptin secretion from PBMCs. The amount of leptin released from PBMCs after the different treatments was enough to stimulate the secretion of IL-1β from monocytes. Targeting leptin sera levels and secretion from PBMCs could represent a new therapeutic strategy to counteract metabolic diseases such as T2DM.

## 1. Introduction

Type 2 Diabetes mellitus (T2DM) is a metabolic disorder where chronic low grade-inflammation and the activation of the innate immunity contribute to the pathogenesis of insulin resistance and pancreatic β cell failure [1]. Inflammation impact on T2DM pathophysiology is even more evident since pro-inflammatory mediators like interleukin-1β (IL-1β) and tumor necrosis factor-α (TNF-α) mediate glucotoxicity effect on pancreatic β-cell dysfunction and increase insulin resistance levels [2,3].

Indeed, T2DM might be considered as an inflammatory disorder, where high nutrient intake may lead to lipid overflow and overload, triggering the activation of the innate immunity, through the toll-like receptors (TLRs) system [4]. In T2DM, insulin resistance decreased glucose-stimulated insulin secretion, and glycosuria might protect against overnutrition by preventing accumulation and overloading of nutrients in different tissues [5]. However, the activation of the innate immune systems happens when these mechanisms are overcome, resulting in the activation of the pro-inflammatory cytokines network. Higher glucose levels can induce the activation of NLRP3 (NLR family pyrin domain containing 3) inflammasome, increasing the synthesis and secretion of IL-1β from different sources such as pancreatic β−cells and macrophages [3]. The high levels of IL-1β inhibit insulin secretion and trigger proapoptotic signaling in pancreatic beta cells [6]. IL-1β and other mediators contribute to the progressive failure of pancreatic beta cells, insulin resistance, and chronic complications of T2DM, such as cardiovascular and cerebrovascular diseases, heart failure, etc. The activation of the innate immunity is implicated in the pathogenesis of insulin resistance and T2DM, also triggering the TLR systems [7,8]. Indeed, different TLR2 and TLR4 polymorphisms increase the risk for T2DM, suggesting a causal relationship between TLR function and T2DM, and its complications [9]. Several studies in mice indicate that TLR2 and TLR4 activation leads to the development of diabetes mellitus through the production of cytokines [10]. Furthermore, T2DM and T1DM patients have higher blood concentrations of TLR ligands with higher activation of the TLR system [11]. Between the TLR ligands, High Mobility Group Box1 (HMGB1) has peculiar properties since modulating insulin resistance and insulin secretion, and it was positively associated with increased body mass index, insulin resistance, and hyperglycemia. HMGB1 is a 25 kDa non-histone DNA binding protein that ensures genome stability and regulates gene transcription preserving DNA interaction with transcription factors [12]. Inflammation triggers HMGB1 release from activated macrophages/monocytes, neutrophils, and natural killer cells between others [13]. HMGB1 can act as a damage-associated molecular pattern molecule (DAMP) and activate the innate and adaptative immunity, driving host inflammatory responses [13]. In adipose cells derived from obese T2DM patients with increased levels of insulin resistance and T2DM, HMGB1 expression is mainly cytoplasmatic and secretion is raised [14]. In the presence of normal blood glucose levels, HMGB1 might stimulate insulin release from pancreatic beta cells, which, differently, is inhibited in the presence of hyperglycemia [14]. High glucose concentrations induce HMGB1 secretion from different sources [15], and this cytokine has been associated with an increased incidence of coronary artery disease and heart failure in T2DM patients [15], proposing an important role of HMGB1 in the pathogenesis of T2DM and its complications. HMGB1 activates innate immune systems by binding specific receptors, primarily TLR2, TLR4, and Receptor for Advanced Glycation Endproducts (RAGE) on human peripheral blood mononuclear cells (PBMCs) [16], suggesting that PBMCs may represent a target for HMGB1. Previously, we found that HMGB1 expression was highly increased in the liver of insulin receptor knockout (IR^−/−^) mice (a model of diabetic ketoacidosis), which developed a severe liver dysfunction associated with increased levels of HMGB1 [17]. This evidence outlines the role of HMGB1 as a mediator of innate immunity in T2DM and its complications. Moreover, HGMB1 promotes interleukin-2 (IL-2) transcription, whose action is mandatory for NK cells activity [18], acting synergistically with other cytokines [19]. IL-2 production is involved in insulin resistance onset, followed by macrophages’ activation and cytokines’ secretion [20]. Recently, T2DM HMGB1 has been reported as a key component in leptin associated inflammatory disorders [21]. Leptin is one of the foremost molecules linking inflammation and defects of glucose metabolism due to its dual role as an endocrine hormone, regulating energy homeostasis and food intake [22] and for its action as an immunomodulator. Leptin influences the secretion of cytokines such as IL-1β, in the acute phase of inflammation, promoting T cells polarization toward pro-inflammatory T helper 1 (Th1) response [23]. Intriguingly, the expression of leptin [24] and leptin receptor [25] was found in PBMCs, suggesting that PBMCs may be implicated in the mechanisms linking immune-inflammation to the modulation of chronic diseases development. Nevertheless, the relationship between leptin release, inflammatory stimuli, such as HGMB1, and their mutual regulation remains to be clarified. We hypothesize that HMGB1 may regulate innate immunity, also affecting leptin release from PBMCs through the activation of the TLRs and/or RAGE. This may open a new scenario in the etiopathogenesis of the innate immunity activation in T2DM.

## 2. Results

### 2.1. PBMCs Pro-Inflammatory Cytokines Secretion in Response to IL-2 and HMGB1

**Figure 1:**
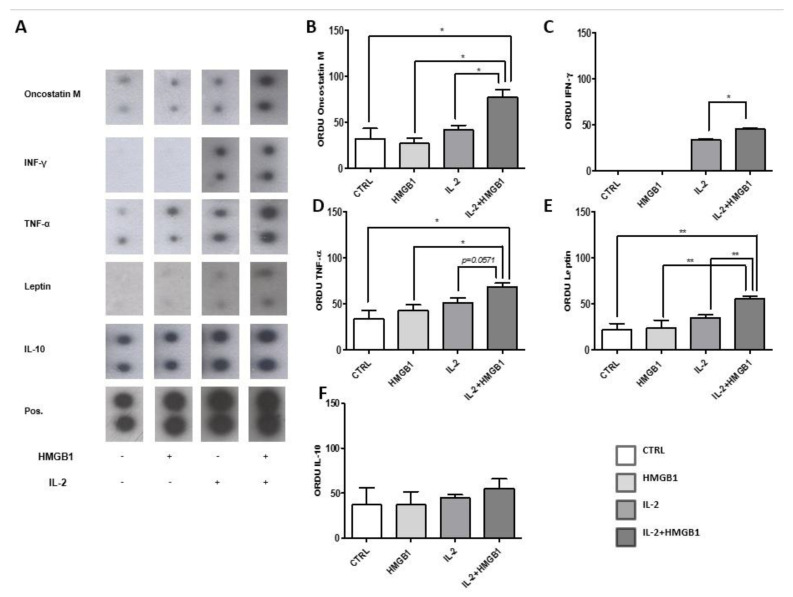
(**A**) Cytokine secretion was evaluated by Western blot assay on PBMCs supernatants. IL-2/HMGB1co-treatment induced a significant increase in the secretion of reported pro-inflammatory cytokines; (**B**) Oncostatin M; (**C**) INF−γ; (**D**) TNF-α; (**E**) Leptin. Treatment with HMGB1 or IL-2 alone did not induce any significant variation in pro-inflammatory cytokines release, except INF-γ, which increased in response to IL-2; (**F**) IL-10 levels were not modified in response to the different treatments; ORDU (optical relative density unit) has been calculated by percentage expressed ratio between optical cytokine absorbance vs. positive control displayed in (**A**). Data are expressed as mean ± SEM for four healthy donors and were analyzed with Wilcoxon–Mann–Whitney U Test (* *p* < 0.05, ** *p* < 0.005). CTRL: control; HMGB1: High Mobility Group Box1; IL-2: Interlukin-2; INF−γ: Interferon−γ; TNF-α: Tumor Necrosis Factor-α; IL-10: Interlukin-10; Pos: positive.

Cytokines’ screening array was performed to analyze the secretion profile of PBMCs in response to HMGB1, IL-2, and combination treatments (Figure 1A). Oncostatin M (OCM) released into the extracellular milieu was increased after IL-2 and HMGB1 treatments, in comparison with IL-2, HMGB1, and serum-containing medium (control-CTRL) (*p* < 0.05) (Figure 1B); OCM secretion has already been reported to increase in response to TLR4 activation [26]. HMGB1 incubation did not lead to interferon-γ (IFN-γ) release from PBMCs; on the contrary, IL-2 stimulation induced the secretion of IFN−γ with a further significant increase when combined with HMGB1 (*p* < 0.05) (Figure 1C), as already reported [27]. Moreover, IL-2 and HMGB1 significantly increased TNF-α secretion compared with HMGB1 alone or control (*p* < 0.05) (Figure 1D), and nearly statistically significant vs. IL-2 (*p* = 0.057) (Figure 1D). In PBMCs, basal leptin release was detected in culture medium with a further significant increase after IL-2 and HMGB1 co-treatment (*p* < 0.005) (Figure 1E), even vs. HMGB1 or IL-2 single treatment and control (*p* < 0.005). Conversely, IL-2 and HMGB1 stimuli did not affect IL-10 (Figure 1F) and IL-4 secretion from PBMCs. Altogether, these results suggested that IL-2 and HMGB1 incubation can stimulate the secretion of leptin and induce a pro-inflammatory response by the activation of PBMCs.

### 2.2. Leptin Secretion from PBMC Subpopulations

**Figure 2:**
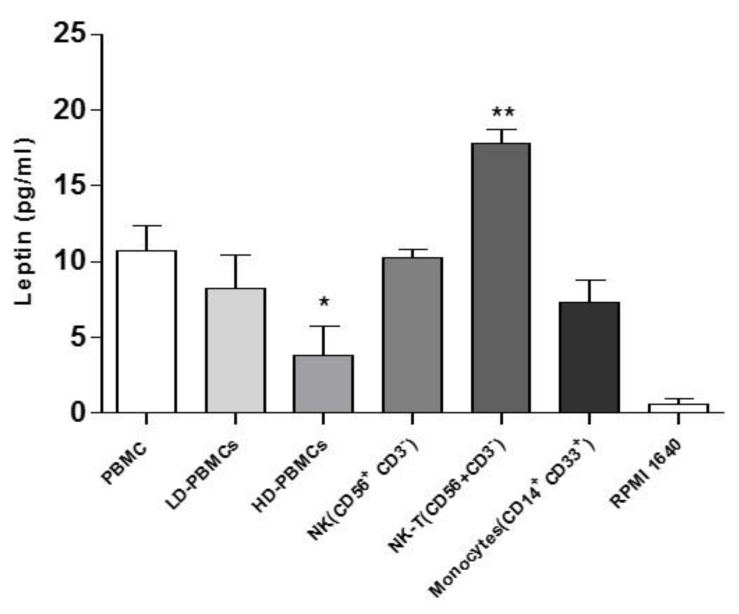
PBMCs basal leptin secretion has been evaluated by ELISA assay. Total PBMCs basal leptin levels were assessed, and no significant variations were observed in LD-PBMCs, NK, and monocytes subpopulations compared to total PBMCs. Basal leptin levels in HD-PBMCs were significantly decreased compared to total PBMCs. NK-T basal leptin levels were significantly higher compared to any PBMCs subpopulation. Each subpopulation has been isolated from three healthy donors’ buffy coat. Data are expressed as mean ± SEM and were analyzed with Wilcoxon–Mann–Whitney U Test (* *p* = 0.05, ** *p* < 0.005). PBMC: peripheral blood mononuclear cells; LD-PBMCs: low density peripheral blood mononuclear cells; HD-PBMCs: high density peripheral blood mononuclear cells; NK: Natural Killer; NK-T: Natural Killer-T.

Leptin secretion in all PBMCs and their subpopulations were confirmed in the basal state (~10 pg/mL) (Figure 2). First, PBMCs were separated into LD (low density)-PBMCs and HD (high density)-PBMCs; then, leptin secretion was measured. LD-PBMCs fraction had comparable levels of leptin secretion as all PBMCs, while a lower amount of leptin release was found from HD-PBMCs fraction (*p* < 0.05). Correspondingly, isolated NK cells and monocytes had similar concentrations of leptin secretion compared to all PBMCs; differently, NK-T cells had significantly higher levels of leptin release (*p* < 0.005) (~17 pg/mL). To avoid biases, completed RPMI1640 medium used to culture PBMCs was utilized as control and, as expected, no leptin levels were detectable.

### 2.3. HMGB1 and IL-2 Co-Treatment Increased Leptin Secretion

**Figure 3:**
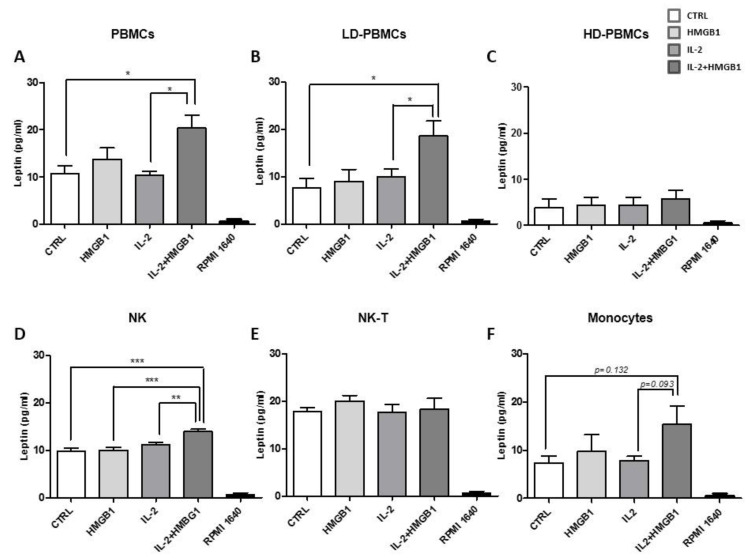
Leptin concentrations were measured by ELISA assay after IL-2 and/or HMGB1 treatments. (**A**) IL-2 and HMGB1 co-treatment induced a significant increase in leptin secretion, while IL-2 or HMGB1 did not cause any raise. PBMCs were sorted using Percoll gradient and separated in low-density PBMCs and high-density PBMCs; (**B**) low-density PBMCs had a significant increment in leptin secretion after IL-2 and HMGB1 co-treatment; (**C**) high-density PBMCs fraction had the lowest secretion of leptin with no enhancement after all treatments; (**D**) isolated NK cells secreted leptin in basal conditions with an additional significant increase in response to IL-2 and HMGB1 co-treatment; (**E**) isolated NK-T cells released the highest amount of leptin without variations after all treatments. (**F**) Isolated monocytes did not show any significant increase in leptin secretion. Leptin was not found in RPMI1640. Each subpopulation has been treated in triplicate, from healthy donors’ buffy coat. Data are expressed as mean ± SEM and were analyzed with a Wilcoxon–Mann–Whitney U Test (* *p* = 0.05, ** *p* < 0.005, *** *p* < 0.0005). CTRL: control; HMGB1: High Mobility Group Box1; IL-2: Interlukin-2; PBMC: peripheral blood mononuclear cells; LD-PBMCs: low density peripheral blood mononuclear cells; HD-PBMCs: high density peripheral blood mononuclear cells; NK: Natural Killer; NK-T: Natural Killer-T.

In all PBMCs, leptin secretion was found to be boosted after IL-2 and HMGB1 co-treatment. We firstly confirmed data on leptin secretion with a quantitative ELISA assay. In IL-2 activated PBMCs, HMGB1 induced a significant increase of leptin secretion (~20 pg/mL) compared with IL-2 treated cells and controls (*p* < 0.05) (Figure 3A). Conversely, any significant variation in leptin secretion was observed after stimulation with HMGB1 or IL-2 incubation. (Figure 3A). Then, in isolated LD-PBMCs, a significant increase in leptin release was observed after IL-2 and HMGB1 co-treatment compared to both controls (*p* < 0.05) and IL-2 incubated cells (*p* < 0.05) (Figure 3B). Differently, HD-PBMCs fraction showed low levels of leptin secretion (~5 pg/mL), without any additional increment in response to IL-2, HMGB1, or their combination (Figure 3C). These results suggest that LD-PBMCs subpopulations are the primary source of leptin secretion when activated in response to a pro-inflammatory stimulus. Therefore, we aimed to identify the LD-PBMCs subpopulations, which might be implicated in the leptin secretion process. Then, in isolated NK cells after activation with IL-2, followed by treatment with HMGB1, leptin secretion was significantly higher vs. control (*p* < 0.0005) (~15 pg/mL), HMGB1 (*p* < 0.0005), and IL-2 (*p* < 0.005) treated cells (Figure 3D). NK-T cells (CD3^+^ CD56^+^) have been isolated and no surge of leptin secretion was detected in response to different treatments (Figure 3E). Afterward, monocytes fraction was purified and IL-2 plus HMGB1 incubation induced a non-significant enhancement of leptin levels compared to control and treatment with IL-2 or HMGB1 (Figure 3F). To avoid possible biases on results from NK, we repeated in vitro experiments by utilizing clonal human NK cell lines (YTS). NK YTS cells secreted leptin in basal conditions, without an increase after HMGB1 or IL-2 treatment alone (Supplementary Figure S1). Conversely, in agreement with previous results, HMGB1 incubation after IL-2 activation showed a significant increase in leptin secretion amount compared to control (*p* < 0.05), HMGB1, or IL-2 (*p* < 0.05) single treatments. Leptin levels were also evaluated in FBS, as well as in RPMI 1640 and RPMI 1640, supplemented with FBS, and no leptin was detected (Supplementary Figure S2). These results suggest that HMGB1 and IL-2 act synergistically as a pro-inflammatory trigger to increase leptin production from PBMCs. To confirm that leptin secretion from PBMCs was strictly associated with a pro-inflammatory stimulus, PBMCs were treated with TNF-α at different concentrations, and a significant increase in leptin secretion was reported vs. control (Supplementary Figure S3). Our data confirmed a pivotal role in leptin secretion during inflammation. To better investigate the biological relevance of NK and NK-T cells in leptin secretion, total PBMCs, depleted of NK and NK-T subpopulation, were incubated with IL-2 and afterwards treated with HMGB1. Then, supernatants were harvested, and leptin levels were assessed with no rise in leptin secretion; afterwards, IL-2 and HMGB1 co-treatment were detected in PBMCs depleted of NK and NK-T (Supplementary Figure S4).

### 2.4. Leptin Secretion from PBMCs Was Mediated by TLR2 and TLR4

**Figure 4:**
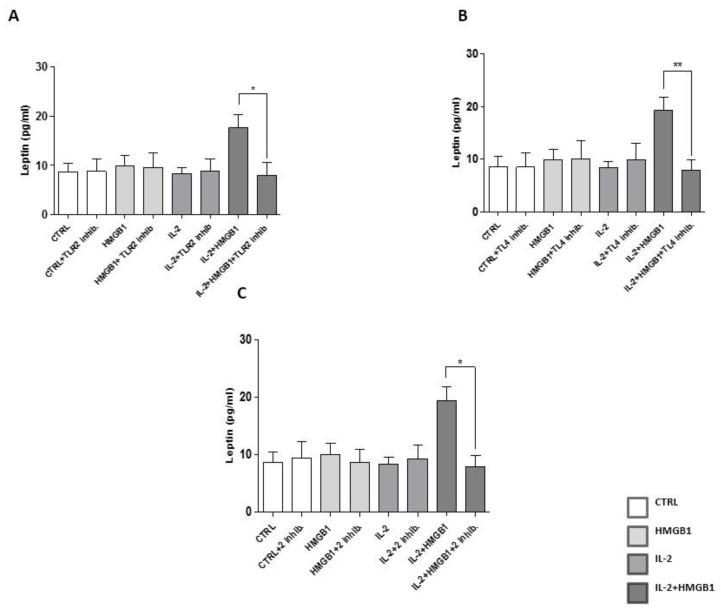
PBMCs leptin secretion were evaluated in the presence of Anti-hTLR2-IgA (TLR2 inhibitor) and/or TAK242 (TLR4 inhibitor) for 24 h; at the end of the treatment, PBMCs were incubated with IL-2 and/or HMGB1. Treatment with anti-hTLR2-IgA (**A**) and TAK242 (**B**) or combined treatment (**C**) significantly blunted leptin secretion in response to HMGB1 and IL-2 co-treatment. PBMCs were obtained from three healthy donors and data are expressed as mean ± SEM and were analyzed with Wilcoxon–Mann–Whitney U Test (* *p* < 0.05, ** *p* < 0.005). CTRL: control; TLR2: Toll Like Receptor 2; HMGB1: High Mobility Group Box1; IL-2: Interlukin-2; TL4: Toll Like Receptor 4.

IL-2 treatment has already been demonstrated to increase TLR2 and 4 surface expression in monocytes [28]. To confirm these data, TLR2 and TLR4 expression were assessed by flow cytometry in isolated PBMCs after IL-2 incubation, finding a significant increase of TLR2 and TLR4 levels (Supplementary Figure S5). Since our data demonstrated that PBMCs’ RAGE levels were not increased by IL-2 priming (Supplementary Figure S6), we then focused our attention on TLR2 and TLR4. To determine the role of TLR2 and 4 in modulating HMGB1 action after IL-2 priming on PBMCs, Anti-hTLR2-IgA, and TAK242, specific inhibitors for TLR2 and TLR4, respectively, were utilized. The release of leptin after treatment with IL-2 and HMGB1 was blunted by co-incubation with Anti-hTLR2-IgA (*p* < 0.05) (Figure 4A), or TAK242 (*p* < 0.005) (Figure 4B) or both inhibitors (*p* < 0.05) (Figure 4C). Our data elucidate a new mechanism through which HMGB1, after IL-2 priming, may modulate leptin secretion from PBMCs, via the arousal of innate immune systems, and activation of a pro-inflammatory response. Next, we hypothesized that activation of TLR2 and TLR4 cell signaling in PBMCs may induce leptin secretion in response to IL-2/HMGB1 co-treatment. To investigate TLR2 signaling, LY294002, an inhibitor of IRF-7 (Interferon Regulatory Factor 7, a key substrate of TLR2) phosphorylation and nuclear translocation was used [29]. In PBMCs treated with IL-2 plus HMGB1, LY294002 was added, as already reported [30]. Afterwards, leptin levels were measured in cell culture medium, finding a significant decrease of leptin secretion vs. PBMCs not incubated with LY294002 (*p* < 0.05) (Figure S7A). To evaluate TLR4 signaling, Necostatin-1 was utilized to inhibit Rip-1, a key component of TLR4 pathway [31], as already described [32]. We found that inhibition of TLR4 signaling significantly reduced leptin section (*p* < 0.005) (Figure S7B) in response to IL-2/HMGB1. These data suggested that leptin secretion in response to IL-2/HMGB1 co-treatment was modulated by TLR2 and TLR4 activation.

### 2.5. IL-1β Secretion Was Induced by Leptin and IL-2/HMGB1 Co-Treatment

**Figure 5:**
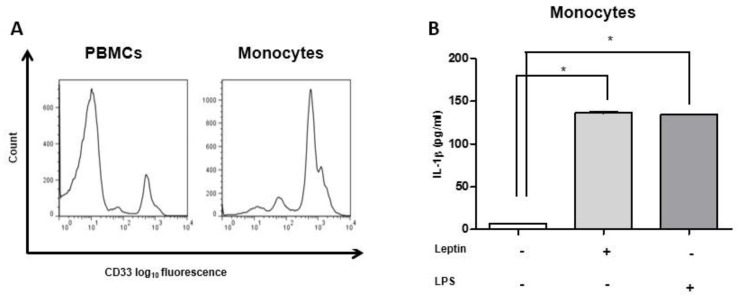
Monocytes were isolated from healthy donors using anti-CD14 magnetic beads; (**A**) Purity was tested with positivity to an anti-CD33 antibody. Monocytes were cultured in RPMI1640 and stimulated with ~20 pg/mL of leptin for 18 h. Afterward, IL-1β secretion was measured; (**B**) ~20 pg/ml of leptin was able to induce a significant release of IL-1β from monocytes, which was comparable to LPS positive control. PBMCs have been treated with LPS 1.5 μg/mL for 18 h. Data are expressed as mean ±SEM for three healthy donors and were analyzed with the Wilcoxon–Mann–Whitney U Test (* *p* < 0.005). PBMC: peripheral blood mononuclear cells; IL-1β: interleukin-1β; LPS: lipopolysaccharide.

Since the concentrations of secreted leptin either in basal conditions or after pro-inflammatory stimuli (IL-2 plus HMGB1) were low, and, to confirm a paracrine action of leptin, CD14^+^ monocytes isolated from PBMCs were treated with leptin at ~20 pg/ml for 18 h. Leptin incubation induced IL-1β secretion from monocytes (*p* < 0.005) similar to the ones obtained in response to lipopolysaccharide (LPS) treatment (*p* < 0.005). LPS treatment was used as a positive control for IL-1β secretion from monocytes (Figure 5B), as already reported [33]. These results allow us to hypothesize a paracrine action, which might be activated in different conditions. Then, isolated total and LD-PBMCs were treated with IL-2, HMGB1, and IL-2/HMGB1 co-treatment, and IL-1β secretion levels were measured, finding a significant increase in IL-1β secretion in PBMCs stimulated with IL-2 plus HMGB1 vs. all other treatments and control (*p* < 0.005) (Figure S8A). Similar results were observed in response to the same treatments in LD-PBMCs with a significant increase of leptin secretion (*p* < 0.0005) (Supplementary Figure S8B). Then, Anti-hTLR2-IgA and/or TAK242 inhibitors of TLR2 and TLR4 signaling, respectively, were added in total PBMCs incubated with IL-2 plus HMGB1, and a significant decrease in IL-1β secretion was detected (*p* < 0.0005) (Supplementary Figure S8C).

### 2.6. Glucose Modulates Leptin Secretion

**Figure 6:**
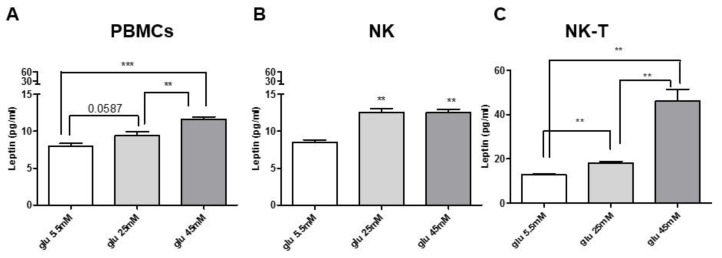
PBMCs, NK cells, and NK-T cells isolated from healthy donors were treated with high glucose concentration for 48 h. Glucose were added every 24 h at 5.5 mM, 25 mM, and 45 mM concentrations. Total PBMCs (**A**) had a significant increase in leptin secretion after 45 mM glucose vs. both 5.5 mM glucose and 25 mM glucose; on the contrary, PBMCs treated with 25 mM glucose did not have a significant increase in leptin secretion (*p* = 0.0587). Isolated NK cells (**B**) displayed a significant increase in leptin secretion in response to 25 mM glucose and 45 mM glucose stimulation compared to 5.5 mM. NK-T cells (**C**) showed a significant increase in leptin secretion in the same experimental conditions as NK cells, with the highest levels in response to glucose 45 mM (~46 pg/mL). Data are expressed as mean ± SEM for three healthy donors and were analyzed with a Wilcoxon–Mann–Whitney U Test (** *p* < 0.005, *** *p* < 0.0005). PBMC: peripheral blood mononuclear cells; NK: Natural Killer; NK-T: Natural Killer-T; glu: glucose.

In order to verify our hypothesis, we treated total PBMCs and NK and NK-T subpopulations with physiological (5.5 mM) and high glucose concentrations (25 mM and 45 mM) for 48 h, as already reported [34,35]. Then, leptin secretion was assessed in PBMCs supernatants. Total PBMCs had an almost significant increase in leptin secretion after incubation with 25 mM glucose vs. 5.5 mM glucose (*p* = 0.0587) and a significant increase after treatment with 45 mM glucose compared to 5.5 mM glucose (*p* < 0.0005) and 25 mM glucose (*p* < 0.005) (Figure 6A). In NK and NK-T cells, leptin secretion was also evaluated in response to hyperglycemia. NK cells had a significant increase in leptin secretion after incubation with 25 mM and 45 mM glucose vs. glucose 5 mM (*p* < 0.005) (Figure 6B). In the same experimental conditions, a significant increase (*p* < 0.005) of leptin secretion was found in NK-T cells. Moreover, NK-T cells have the highest increase in leptin secretion in response to 45 mM glucose, which was also higher vs. glucose 25mM (*p* < 0.005) (Figure 6C).

### 2.7. Insulin Treatment and Leptin Secretion

**Figure 7:**
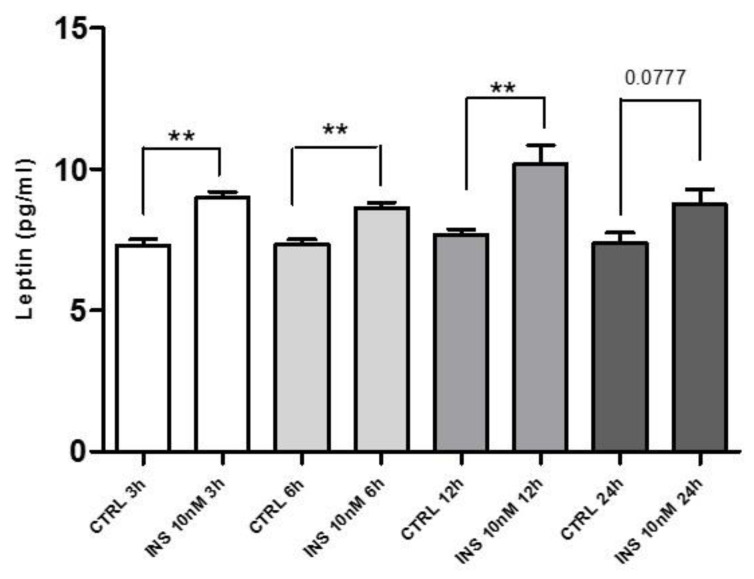
PBMCs were isolated from healthy donors’ buffy coat, washed twice in PBS, and then treated with high insulin levels (10 nM) for 3 h, 6 h, 12 h, and 24 h. Cells supernatant were used to evaluate leptin secretion, which was compared to the untreated samples. A significant increase in leptin secretion was detected after 3 h, 6 h, and 12 h of insulin treatment, but a not significant increase was found after 24 h. Data are expressed as mean ± SEM for three healthy donors and were analyzed with Wilcoxon–Mann–Whitney U Test (** *p* < 0.005). CTRL: control; INS: insulin.

PBMCs were treated with high insulin concentrations 10nM as already reported [36,37], since insulin levels are usually higher at the beginning of T2DM. Leptin secretion was measured in response to insulin time-course treatment (3 h, 6 h, 12 h, and 24 h). We found that leptin secretion was significantly increased (*p* < 0.005) after 3 h, 6 h, and 12 h of insulin treatment. Conversely, after 24 h of insulin incubation, there was a not significant increase of leptin secretion. These data demonstrated that insulin treatment increased leptin secretion from PBMCs (Figure 7).

### 2.8. IL-2 and HMGB1 and Leptin Secretion in T2DM Patients’ PBMCs

**Figure 8:**
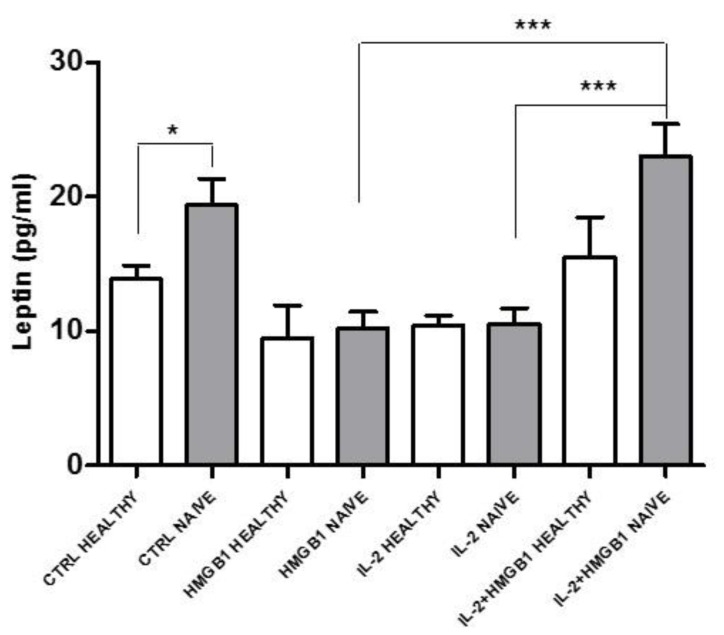
PBMCs were isolated from healthy donors and newly diagnosed T2DM patients (naïve). Isolated PBMCs have been washed twice, incubated with HMGB1 or IL-2 and IL-2 plus HMGB1 (PBMCs were incubated with IL-2 for 72 h and then treated with HMGB1 for the following 48 h). At the end of treatments, leptin secretion was evaluated on serum supernatants in both healthy and naïve PBMCs donors. IL-2/HMGB1 coupled treatment increased leptin secretion in naïve PBMCs compared to both IL-2 or HGMB1 single treatment, similar results were obtained in healthy donors’ PBMCs. The amount of leptin released by naïve PBMCs was found to be significantly increased compared to healthy PBMCs, especially in basal conditions. Data are expressed as mean ± SEM and were analyzed with Wilcoxon–Mann–Whitney U Test (* *p* < 0.05, *** *p* < 0.005, *n* = 5 each group). CTRL: control; HMGB1: High Mobility Group Box1; IL-2: Interlukin-2.

To corroborate the role of leptin secretion by PBMCs in T2DM, we evaluated leptin release from T2DM patients’ isolated PBMCs in basal conditions and after IL-2 and HMGB1 co-treatment. Since several hypoglycemic drugs have anti inflammatory pleiotropic effects [38,39], we chose to enroll patients with a neodiagnosis of T2DM (naïve) without any hypoglycemic treatment. PBMCs isolated from T2DM patients displayed a higher leptin secretion in response to IL-2 and HMGB1 co-treatment vs. IL-2 (*p* < 0.005) and HMGB1 (*p* < 0.05) single treatments (Figure 8). Moreover, we found that T2DM naïve patients’ PBMCs showed significant higher levels of leptin secretion vs. PBMCs of healthy subjects in basal conditions (*p* < 0.05). However, non-significant differences were observed in response to IL-2 or HMGB1 single treatments and to IL-2 and HMGB1 co-treatment, between the two study groups (Figure 8). These data demonstrated that PBMCs of naïve T2DM had higher levels of leptin secretion at least in basal conditions and an increased susceptibility to a pro-inflammatory stimulus.

## 3. Discussion

Leptin is an adipocytokine primarily produced by adipocytes, which, through its pro-inflammatory properties, is implicated in promoting several pathological conditions, such as T2DM, obesity, and cancer [40,41]. Therefore, leptin might be considered a pro-inflammatory cytokine given that it contributes to the ‘low-grade inflammatory state’ in T2DM patients and overweight and obese individuals with high levels of insulin resistance [42]. The tissues and serum expression levels are also regulated by inflammatory stimuli, such as LPS [43], even if the mechanisms underlaying leptin-mediated inflammation damage are still vague. However, leptin may expand cancer cells through the production of cytokines by macrophages [44,45] and also upregulate the pro-inflammatory cytokines such as TNF-α and IL-6 and stimulated the macrophage function [46]. In the present study, we confirmed that PBMCs can secrete leptin after pro-inflammatory stimuli [47], and the highest secretion levels of leptin were obtained from NK-T cells, in particular in response to high glucose concentrations. Moreover, PBMCs treated with IL-2 and HMGB1, a pro-inflammatory stimulus, led to a significant increase of leptin secretion. Here, we also showed that NK and, in part, monocytes were the PBMCs subpopulations releasing the higher amount of leptin in response to IL-2 and HMGB1 stimuli by the activation of the innate immunity, through the toll-like receptor systems (TLR2 and 4). Furthermore, we documented that TNF-α can also increase the secretion of leptin, suggesting that pro-inflammatory cytokines/stimuli might modulate leptin secretion from PBMCs. These data suggest that leptin, besides its known endocrine and pro-inflammatory actions, may modulate the activation of PBMCs and immunity response through a paracrine action on PBMCs. This process may further increase in response to pro-inflammatory stimuli. The link between leptin action in mediating the development of chronic metabolic diseases and inflammation is very complex. Flux nutrients’ excess may activate TLR2, TLR4, and other pattern recognition receptors (PRRs) inducing a chronic inflammation state, determined by an altered profile of cytokine production [43]. In the present study, we reported that the activation of TLR2 and 4 is pivotal to increase the secretion of leptin from PBMCs after stimulation by IL-2 and then HMGB1. The role of HMGB1 as a mediator in the secretion of pro-inflammatory cytokines after different stimuli such as hyperglycemia has already been reported in SV40 MES 13 cells by activating the NF-κB pathways [48]. Then, leptin may directly stimulate monocytes/macrophages to produce other pro-inflammatory cytokines, such as IL-1β, with a paracrine action, which next may stimulate and activate T-cells to synthesize IL-6 [49]. Similarly, HMGB1 can be secreted from adipose tissue [13] and acts as an innate alarmin to induce the activation of resident immune cells [11,12]. Furthermore, in agreement with previous findings, Dasu et al. [4] showed that circulating levels of HMGB1 were significantly increased in T2DM patients. PBMCs were pre-incubated with IL-2 and then stimulated with HMGB1, since IL-2 can activate different PBMCs functions [50] and increase the expression of HMGB1 receptor TLR4 [28]. We showed that IL-2 priming of PBMCs before HMGB1 treatment was mandatory since HMGB1 single incubation was not able to increase the inflammatory process, as already reported [51]. IL-2 has been directly positively correlated with increased levels of insulin resistance [20], and higher sera soluble IL-2 receptor (sIL-2r) levels were observed in T2DM patients as well [52]. These data are particularly significant since sIL-2r/IL-2 interaction was found to increase the activity of IL-2 boosting its inflammatory signal [53]. Based on these findings, we chose to treat PBMCs with HMGB1 in combination with IL-2 to reproduce a microenvironment similar to the inflammatory-related conditions potentially present in metabolic diseases such as T2DM. In IL-2 activated PBMCs, HMGB1 induced INF-γ, TNF-α, and OCM secretion, while no significant release was found after IL-2 or HMGB1 single treatment. Therefore, we hypothesized that an IL-2 and HMGB1 synergic action mainly affects Th-1 with no effect on Th-2 response since no significant changes in IL-10 and IL-4 secretion were observed in response to different treatments.

A relationship between HMGB1, IFN-γ, and TNF-α was already shown in T2DM animal models, as a modulatory mechanism of immunity and inflammation [54]. Furthermore, in T2DM, OCM blood concentrations were increased, and its release was due by F4/80^+^ macrophages infiltrating white adipose tissue [55]. The augmented levels of INF-γ were linked to higher levels of insulin resistance [56], and HMGB1 and IL-2 co-treatment could increase the release of IFN-γ in PBMCs and NK-cells, as already reported [27].

Then, in the present study, in basal conditions, NK-T subpopulation had the highest levels of leptin secretion; nevertheless, since the amount of circulating NK-T cells represents about 0.2% of circulating T cells [57], basal leptin secretion might not have physiological relevance. The secretion of leptin by PBMCs was significantly increased after HMGB1 and IL-2 co-treatment, mainly from NK cells, suggesting that, after pro-inflammatory stimulus, leptin secretions can increase from NK cells. We substantiated this speculation proving that NK and NK-T depleted PBMCs did not increase leptin levels after IL-2 and/or HMGB1 treatments. Then, dysregulation of NK activity may affect leptin secretion after pro-inflammatory stimuli.

Intriguingly, a relationship between low-grade inflammation, adipokines, and NK cell number and activation were already suggested [58]. Circulating NK cells are higher in T2DM patients with or without colon cancer, with a NK reduced cytotoxic activity in T2DM with colon cancer, demonstrating that the risk of developing a carcinogenic process in T2DM patients is associated with a reduced NK cell function [59]. The rise in leptin secretion, with a subsequent enhancement of pro-inflammatory response, coupled with NK cell loss in their cytotoxic activity possibly being directly associated with the pathogenesis of T2DM and its complications. Indeed, it has been well established that NK cells are pivotal for the immune surveillance necessaries to protect against oncological diseases [49]. Furthermore, the incidence of mortality due to cancer in T2DM patients is higher compared to non-diabetic subjects [60]. This worse outcome might be explained, with an NK loss of function in T2DM patients. More epidemiological studies are needed to confirm this finding. This particular pro-inflammatory action of HMGB1 can be mediated by the activation of innate immune systems through the activation of PRRs. Indeed, TLR2 and 4 have been reported to be the most abundant among the TLRs in PBMCs [61]. Since we demonstrated that inhibition of TLR2 and 4 in PBMCs blunted leptin secretion in response to HMGB1, this cytokine might be considered a new target to reduce pro-inflammatory response in T2DM and its related conditions. The amount of secreted leptin from PBMCs activated IL-1β release, a macrophage inflammatory marker [62], confirms its biological effect. We may speculate that leptin can act in a paracrine way to increase IL-1β secretion affecting pancreatic β cell failure [63]. In adipocyte cells, insulin may induce leptin secretion and mRNA expression through a different number of potential mechanisms even if strong evidence outlines either an action mediated by cAMP (Cyclic adenosine monophosphate) [64], or through glucose metabolism and its incorporation into lipid [65]. Glucose may also modulate leptin secretion through a cAMP dependent mechanism at a concentration of 5.5 mM [64]. cAMP/PKA (Protein Kinase A) pathway has been proposed as a potential therapeutic target against metabolic disease, as T2DM [66]. In our experimental model, the inhibition of PKA by KT5720 significantly reduces insulin induced leptin secretion, and it is possible to speculate that some hypoglycemic agents can decrease PBMC leptin secretion in response to high insulin levels. Differently, in PBMCs treated with high glucose concentrations and KT5720 leptin secretion was slightly increased. These results outline the possibility of a different action of insulin and glucose as leptin secretagogues in PBMCs. This point deservers further study. Then, we evaluated if the T2DM milieu with high insulin or glucose concentrations may stimulate leptin secretion from PBMCs, inducing a pro-inflammatory activation. Leptin secretion was increased in response to both treatments. Insulin stimulation of leptin secretion decreased after 24 h of incubation, and the high glucose concentrations induced an increase of leptin secretion in PBMCs and in NK and NK-T subpopulations, and the latter had the highest levels of leptin secretion. Finally, we reported that PBMC function may be modulated in response to ‘metainflammation’—then, in the presence of innate immune systems activation and glucose dysmetabolism, affecting, in particular, NK and NK-T cells subpopulations. We may also speculate that reduced leptin secretion from adipose and/or circulating immune cells may represent a new target to treat inflammatory and metabolic diseases. Further investigations are mandatory to confirm this hypothesis. Finally, we analyzed the response of PBMCs extracted from naïve T2DM patients in basal conditions and in response to treatments with our pro-inflammatory stimuli. Leptin secretion levels increased in T2DM patients in the basal state. This could be explained by the higher levels of pro-inflammatory state in naïve T2DM patients compared to healthy controls. However, in response to IL-2 and HMGB1 co-treatment compared to healthy subjects, only a trend of increase in leptin secretion is present, since statistical significance was not reached. It may be possible to speculate that a low number of diabetic patients and heterogeneity of T2DM disease were factors that impact the statistical analysis. Moreover, it is also possible to hypothesize that T2DM naïve subjects have a higher inflammatory state compared to healthy subjects, which may contribute, at least in part, to increased release of leptin in their basal state. This pro-inflammatory condition may also partially explain the lack in the increasing leptin release in this cohort after pro-inflammatory stimuli, suggesting that, to reach further secretion of leptin, the threshold of these patients may be higher compared to healthy subjects. However, further clinical studies are needed in large populations to confirm the clinical significance of these data and to characterize the phenotype of T2DM patients affected by this defect.

We need to acknowledge some limitations in this study. Firstly, we are aware that the HMGB1 dosage used in this study was high; anyhow, we aimed to emulate acute inflammation. Further studies will be required to determine the HMGB1 minimum effectiveness dose. Secondarily, PBMCs priming can be performed with other Th-1 cytokines to improve or decrease leptin secretion from stimulated PBMCs. In conclusion, we demonstrated a new action of HMGB1 in the presence of IL-2, which, activating the TLR systems in PBMCs, modulates the secretion of different cytokines and leptin, mainly from NK cells. This could enhance the pro-inflammatory response increasing the secretion of IL-1β from monocytes (Supplementary Figure S9). Furthermore, in the presence of high insulin and glucose concentrations PBMCs, mainly NK and NK-T subpopulations may be activated, and leptin secretion was increased. These data suggested that PBMCs may be activated in response to “metainflammation” and leptin secretion might indicate the severity of the dysmetabolism. Further studies are mandatory to confirm these results. This finding might lead to the final discovery of new potential targets for T2DM therapy or innovative therapeutic approaches or strategies (NK and NK-T subpopulations).

## 4. Materials and Methods

### 4.1. Isolation of PBMCs and Their Subpopulations

PBMCs were isolated from several healthy donors’ buffy coat, who signed an informed consent and five naïve T2DM (whose consent was waived because T2DM naïve blood samples used for this study were, ‘waste of the sample examined’ not used for the dosage of biochemical analytes) patients’ blood samples by Ficoll-Hypaque (GE-Healthcare, Little Chalfont, UK) density separation and their subpopulations were isolated by a discontinuous Percoll (GE-Healthcare, Little Chalfont, UK) gradient, as already reported [67]. PBMCs were added to discontinuous gradient and centrifuged at 300× *g* for 50 min to separate two density phases: low-density PBMCs (LD-PBMCs) at the interface between the two different Percoll phases, while high-density PBMCs (HD-PBMCs) at the bottom of the tube. LD-PBMCs fraction contains B cells, monocytes, NK cells, and NK-T cells, while HD-PBMCs include T-Cells and blood-red cells [67]. The University of Rome Tor Vergata Ethic committee approved the PBMC extraction protocol for research purposes and all performed experiments (protocol code: 254.19).

### 4.2. NK Cells Isolation

NK cells were isolated using anti-CD56 magnetic beads separation (Miltenyi Biotech, Bergisch Gladbach, Germany). LD-PBMCs and anti-CD56 magnetic beads were allowed to interact in a buffer solution (PBS + 0.5% BSA), and the cell suspension was passed through a separation column. NK cells retained in the column were mechanically eluted. To determine purity, NK cells were stained with APC-anti CD56 (BD, Franklin Lakes, New Jersey, NJ, USA) and PE-anti CD16 (BD). After 20 min of incubation at +4 °C, stained cells were analyzed with FACSCalibur cytometer, and purity was assessed using Cellquest Pro software (BD, v. 5.1).

### 4.3. NK-T Cells Isolation

NK-T cells isolation was performed using the CD3^+^CD56^+^ NK-T isolation (Miltenyi Biotech) kit. LD-PBMCs were labeled with magnetic beads provided in the kit; magnetic separation depleted the non-NK-T population. Positive selection of NK-T cells was performed by incubation with CD56 magnetic beads. NK-T purity was checked by flow cytometry analysis using antiCD3-CY5 (BD) and antiCD56-APC cells (BD).

### 4.4. CD14 Monocytes Isolation

CD14 monocytes were isolated from LD-PBMCs by incubation with anti-CD14 magnetic beads (Miltenyi Biotech). LD-PBMCs were mixed with anti-CD14 magnetic beads suspension and allowed to interact for 15 min at +4 °C. Cells and anti CD14 magnetic beads mixture were passed through magnetic separation columns (Miltenyi Biotech) and CD14^+^ cells were eluted. CD14^+^ purity was confirmed by flow cytometry analysis using APC-conjugated anti-CD33 (BD).

### 4.5. Cell Culture

PBMCs, LD-PBMCs, HD-PBMCs, NK, NK-T cells, monocytes, and YTS cells were grown in Serum Containing Medium (SCM) RPMI 1640 plus 10% Foetal Bovine Serum (FBS) (Corning, New York, NJ, USA) and penicillin/streptomycin 5% solution (Thermofisher Scientific, Waltham, MA, USA) and activated with IL-2 (Preprotech, London, UK) (200 U/mL) for 72 h at 37 °C and 5% CO_2_, and, afterwards, incubated with human HMGB1 (Sigma Aldrich, Saint Louis, MO, USA) (2 μg/mL) for 48 h at 37 °C, as already reported [68]. TLR2 and TLR4 inhibition were performed using an anti TLR2 blocking antibody (10 μg/mL) and TAK242 (100nM) (Invivogen, San Diego, CA, USA), as already reported [69]. Inhibitors were added 24 h before HMGB1 treatment, during IL-2 incubation.

### 4.6. Western Blotting Analysis

Cytokine levels were evaluated using Human Cytokine array-3 (Raybiotech, Peachtree Corners, GA, USA). PBMCs supernatants were incubated on antibody spotted membranes and then washed and incubated with a biotin-labeled antibody. Cytokine revelation was performed with horseradish peroxidase-labeled streptavidin incubation and determined by Optical Relative Density Units (ORDU) calculation. ORDU for each cytokine was obtained by calculating its ratio with ORDU of positive control provided by the kit.

### 4.7. Enzyme-Linked Immunosorbent Assay

Leptin secretion was measured with an enzyme-linked immunosorbent assay (ELISA) (Raybiotech) and detected with a biotinylated anti-leptin antibody, followed with streptavidin incubation. The IL-1β amount secreted by CD14^+^ monocytes was determined using the IL-1β ELISA kit (R&D Systems, Minneapolis, MN, USA). Samples were read on 450 nm with Victor multilabel counter (PerkinElmer, Waltham, MA, USA), and values were obtained by interpolating the results with a standard curve provided in both detection kits. Data obtained by an ELISA assay were normalized by cell number count before and after treatment.

### 4.8. Statistical Analysis

Statistical analysis was performed using the software program SPSS, v. 16.0 (SPSS Inc., Chicago, IL, USA). Data were expressed as mean ± standard error of the mean (SEM) and analyzed according to the non-parametric Wilcoxon–Mann–Whitney U Test. Results were considered significant when the *p*-value was <0.05.

## Supplementary Materials

Supplementary materials can be found at https://www.mdpi.com/article/10.3390/ijms22157988/s1. Figure S1: YTS cell line significantly increased leptin release after HMGB1/IL-2 stimuli. Figure S2: cell media did not interfere with leptin secretion measurement. Figure S3: TNF-α treatment increased leptin secretion in PBMCs. Figure S4: IL-2 and HMGB1 proinflammatory treatment failed to increase leptin secretion in CD56 depleted PBMCs. Figure S5: IL-2 treatment increased TLR2 and TLR4 expression in PMBCs. Figure S6: IL-2/HMGB1 proinflammatory treatment did not modify RAGE levels in PBMCs. Figure S7: Leptin secretion is regulated by TLR2 and TLR4 pathway. Figure S8: IL-2/HMGB1 co-treatment increased IL-1β secretion in total and LD-PBMCs with a TLR2-TLR4 dependent mechanism. Figure S9: schematic representation of Leptin secretion in response of IL-2/HMGB1 in PBMCs and their subpopulation.
