# Supplementary material for: Activation of Peripheral Blood Mononuclear Cells and Leptin Secretion: New Potential Role of Interleukin-2 and High Mobility Group Box (HMGB)1
Source: Int J Mol Sci. 2021 Jul 26;22(15):7988. doi: 10.3390/ijms22157988 (PMC8347813; doi:10.3390/ijms22157988)
Supplement: Supplementary file 1 [file ijms-22-07988-s001.zip › ijms-1105038-supplementary.pdf]

International Journal of
Molecular Sciences

MDPI

Supplementary Results

1. S1 YTS Cell Line Significantly Increased Leptin Release after HMGB1/IL-2 Stimuli

In order to avoid false positives in leptin secretion analysis due to NK cell isolation, we aimed to confirm our data using an NK cell line, such as YTS. In Supplementary Figure S1, single treatment with HMGB1 or IL-2 did not increase basal leptin secretion levels by YTS vs. CTRL (not treated PBMCs) On the other hand, IL-2 and HMGB1 co-treatment induced a significant increase in leptin secretion vs. CTRL ($p < 0.05$), HMGB1 ($p < 0.05$) and IL-2 ($p < 0.05$), revealing a synergistic leptin release mechanism in response to our stimuli. To avoid any bias in leptin determination due to cell culture medium background noise, leptin levels were measured in RPMI1640 showing no detectable leptin quantity. Similarly to NK cells, basal leptin levels were around 10 pg/mL.

2. S2 Cell Media Did Not Interfere with Leptin Secretion Measurement

Leptin amount detected after our treatments was on the order of picograms. To be sure that neither RPMI1640 nor FBS serum could affect leptin measurement after our stimuli, we aimed to determine background noise levels in any cell culture medium used for PBMCs and their subpopulations. We then determine leptin amount in raw RPMI 1640 cell medium, in fetal bovine serum (FBS) alone and in RPMI1640 supplemented with FBS. Supplementary Figure S2 and no leptin amount were detectable. Taken together, these data demonstrated that no background noise was detectable in cell media used for these experiments.

3. S3 TNF-α Treatment Increased Leptin Secretion in PBMCs

We demonstrated that leptin secretion was associated with a HMGB1 plus IL-2 pro-inflammatory stimuli. Next, to confirm that leptin secretion was actually associated with an inflammatory state, PBMCs were treated with TNF-α at various dosages. Then, 24 h of TNF-α treatment at 0.1 ng/mL significantly increases leptin ($p < 0.05$) secretion compared to CTRL cells. Similarly, higher TNF-α dosage at 10 ng/mL induced a significant increase in leptin secretion compared to CTRL cells ($p < 0.0005$) after 24 h of incubation. Taken together, these data suggested that leptin secretion from PBMCs were modulated with pro-inflammatory stimuli.

4. S4 IL-2 and HMGB1 Proinflammatory Treatment Failed to Increase Leptin Secretion in CD56 Depleted PBMCs

To confirm the role of NK and NK-T in the modulation of leptin secretion in basal state and in response to pro-inflammatory stimuli, healthy donors PBMCs were isolated and depleted of CD56 subpopulation by negative magnetic selection from PBMCs. Afterwards, CD56 depletion NK and NK-T subpopulations were removed from total PBMCs. Then, CD56 depleted PBMCs were incubated with IL-2 and/or HMGB1. After treatments, no significant increment of leptin secretion was found. Our data demonstrated the biological relevance of these subpopulations in increasing leptin secretion after pro-inflammatory stimuli.

5. S5 IL-2 Treatment Increased TLR2 and TLR4 Expression in PMBCs

PBMCs were treated with IL-2 to quantify the expression of TLR2 and TLR4. The amount of TLR2 and TLR4 was analyzed by flow cytometry with a suitable surface staining antibody for TLR2 and/or TLR4. We found a modest upward tendency of $TLR2^{+}$ (A), $TLR4^{+}$ (B) positive cells and a significantly higher amount of $TLR2^{+,}$ $TLR4^{+}$ double positive stained PBMCs (C). Mean Fluorescence intensity (MFI) of both TLR2 and TLR4 also increased after IL-2 priming (D). These results further confirmed that IL-2 increased the expression of TLR-2 and 4 in PBMC plasma membrane, as already reported (28). These data

can explain the higher susceptibility of PBMCs to HMGB1 treatment after IL-2 priming, since TLR2 and TLR4 are specific HMGB1 receptors.

6. S6 IL-2/HMGB1 Proinflammatory Treatment Did Not Modify RAGE Levels in PBMCs.

IL-2 priming on PBMCs has been demonstrated to increase TLR4 and TLR2 receptor expression on PBMCs. Since the Receptor of Advanced Glycation End products (RAGE) is an HMGB1 receptor as well, RAGE levels were determined on PBMCs in basal conditions and after treatments with IL-2 and/or HMGB1. Our data demonstrated that IL-2 priming was not able to increase RAGE levels since mean fluorescence levels were not significantly higher compared to CTRL cells. Similarly, HMGB1 treatment alone did not augment RAGE levels on PBMC surface. Furthermore, HMGB1 administration followed by IL-2 priming on PBMCs failed to increase RAGE levels. Our data suggested that RAGE was not implicated in HMGB1 related pro-inflammatory response.

7. S7 Leptin Secretion Is Regulated by TLR2 and TLR4 Pathways

In order to investigate signaling pathway that can regulate IL-2/HMGB1 induced leptin secretion, we perform a selective inhibition of both TLR2 and TLR4 signaling by adding LY294002 (30μM) (TLR2 signal inhibitor by blocking IRF7)(A) and Necrostatin-1(100 μM)(B) (TLR4 signal inhibitor by blocking RIP-1) for 48h. Our data demonstrated that selective inhibition of both TLR2 and TLR4 intracellular signal pathways was able to significantly reduce leptin secretion amount in response to IL-2/HMGB1 co-treatment. These data confirmed that leptin secretion in PBMCs is regulated by TLR2 and TLR4 signaling pathways.

8. S8 IL-2/HMGB1 Co-Treatment Increased IL-1β Secretion in Total and LD-PBMCs with a TLR2-TLR4 Dependent Mechanism

IL-1β secretion has been evaluated in response to IL-2 and/or HMGB1 single or double treatment to further investigate the pro-inflammatory response. Total LD-PBMCs have been isolated and next incubated with the different treatments. Afterwards, cells were harvested and supernatants were collected to quantify IL-1β concentrations in response to the different treatments. In total PBMCs, IL-2/HMGB1 co-treatment significantly increased IL-1β secretion vs. treatments with IL-2 or HMGB1 and control cells (CTRL). Similarly, in isolated LD-PBMCs, IL-2/HMGB1 co-treatment significantly improved IL-1β secretion vs. treatments with IL-2 or HMGB1 and CTRL. Next, anti-hTRL2-IgA and/or TAK242 were used to inhibit TLR2 and TLR4, respectively, in PBMCs treated with IL-2 plus HMGB1. Then, IL-1β concentrations were measured, finding a significant decrease of IL-1β secretion in the presence of anti-hTRL2-IgA ($p < 0.0005$) and TAK242 ($p < 0.0005$) or both inhibitors ($p < 0.0005$) vs. cells without inhibitors.

Supplementary Figures

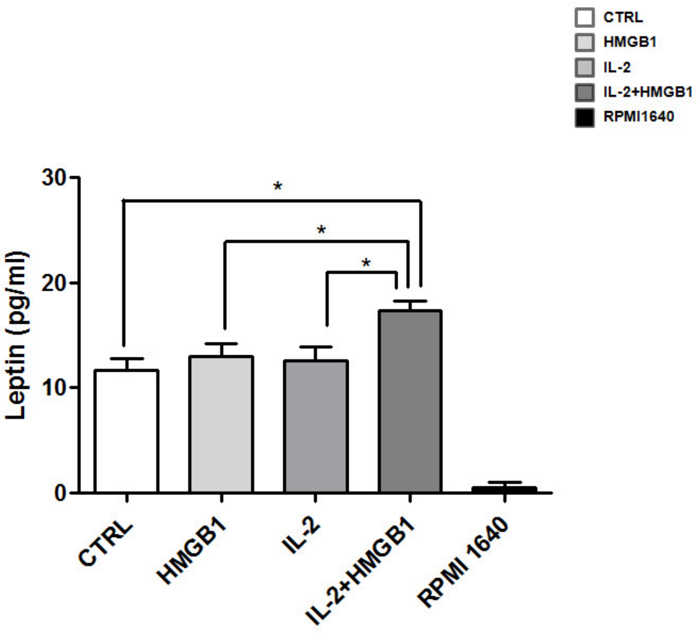

Figure S1. YT-S released leptin after IL-2 and HMGB1 co-treatment. Leptin secretion was quantified in the YT-S NK cell line and IL-2, and HGMB1 co-treatment significantly increased leptin secretion vs. control (CTRL), HMGB1 and IL-2. To avoid bias in leptin measurements due to sample medium interference, leptin levels were measured in RPMI 1640 resulting undeterminable. Data are expressed as mean ±SEM for six replicates and analyzed with the Wilcoxon–Mann–Whitney U Test (* $p < 0.05$).

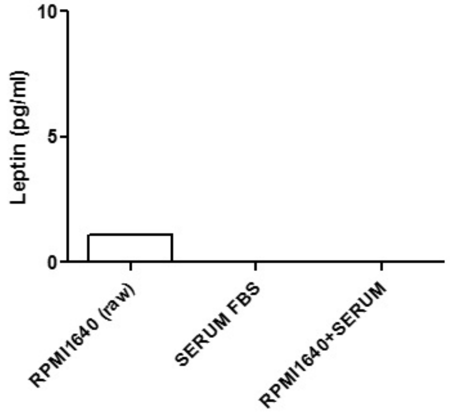

Figure S2. Leptin background noise in Fetal bovine serum (FBS), RPMI1640 raw and FBS supplemented RPMI1640: Samples' aliquots of Fetal Bovine Serum (FBS), RPMI1640 raw cell medium, and RPMI 1640 cell medium supplemented with 10% FBS were screened for leptin background. Leptin was unrevealed testing FBS or RPMI supplemented with FBS. The RPMI medium showed an undetectable amount of Leptin. Experiments were performed in triplicate.

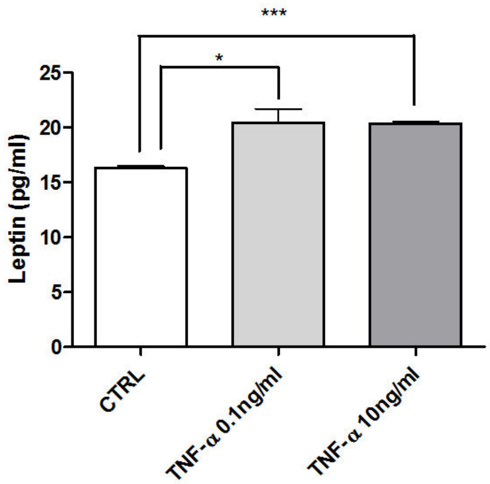

Figure S3. Leptin levels were measured by the ELISA assay after TNFα treatment. Isolated PBMCs were treated with 0.1 ng/mL or 10 ng/mL TNFα dosage, and leptin levels were detected in PBMC supernatant. Low TNFα dosage has been reported to induce a significant increase of leptin secretion from human healthy PBMCs vs. CTRL. No significant differences have been highlighted comparing low and high TNFα dosage treatments. Experiments have been performed in triplicate, from healthy donors' buffy coat. Data are expressed as mean ± SEM and analyzed with a Wilcoxon–Mann–Whitney U Test (* $p = 0.05$, *** $p < 0.0005$).

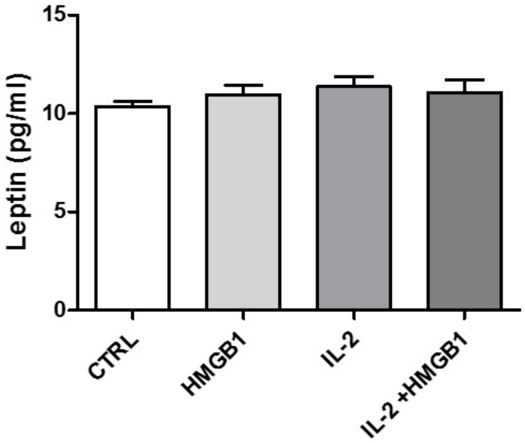

Figure S4. Total PBMCs were depleted from CD56 subpopulation and leptin levels were measured by ELISA assay after IL-2 (200 U) for 72 h and HMGB1 treatment (1 µg/mL) for the next 48 h. No significant variation in leptin secretion was detected after treatments compared to control. Experiments were performed in triplicate, from healthy donors' buffy coat. Data are expressed as mean ± SEM and analyzed with a Wilcoxon–Mann–Whitney U Test.

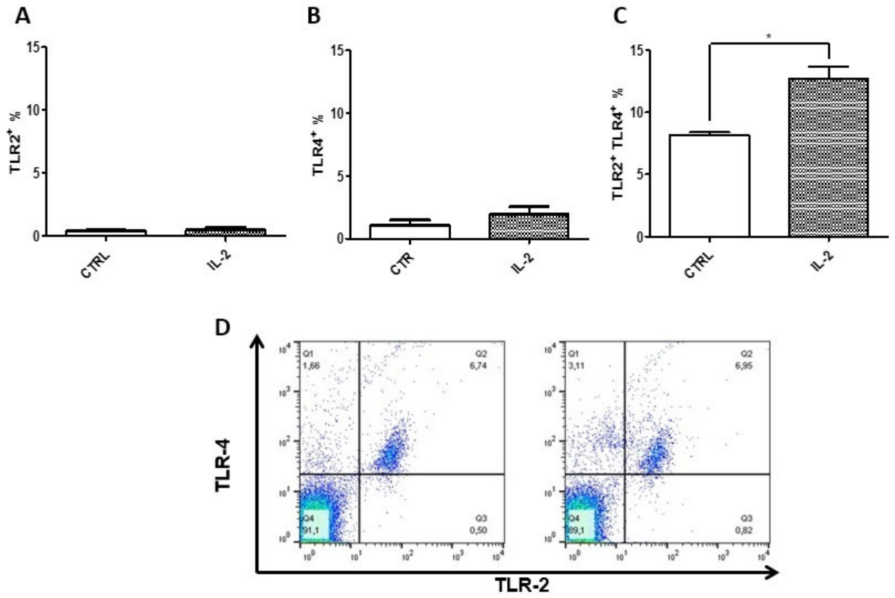

Figure S5. TLR2 and TLR4 amount increased after IL-2 priming. IL-2 (200 U/mL) for 72 h was used to stimulate isolated PBMCs. After treatment, PBMCs were washed twice in PBS and TLR2 and TLR4 percentage were evaluated by flow cytometry after a suitable antibody incubation. IL-2 priming significantly increased TLR2 and TLR4 double positive PBMCs (**C**) percentage, while a non-significant upward trend was reported for TRL2 (**A**) and TLR4 single positive cells; (**B**) experiments were performed in triplicate, from healthy donors' buffy coat. Data are expressed as mean ± SEM and analyzed with Wilcoxon–Mann–Whitney U Test (* $p < 0.05$).

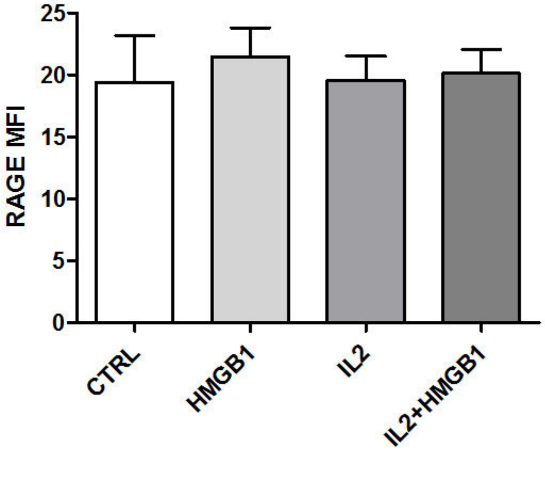

Figure S6. PBMCs were isolated and treated with IL-2 (200 U) for 72 h and HMGB1 (1 µg/mL) for 48 h and then RAGE levels were measured in terms of mean fluorescence intensity (MFI) on PBMC surface by flow cytometry. IL-2 priming was not able to increase RAGE levels compared to SCM and, similarly, neither HMGB1 treatment alone nor IL-2/HMGB1 combined treatment increased RAGE expression on the PBMC surface. Experiments were performed in triplicate, from healthy donors' buffy coat.

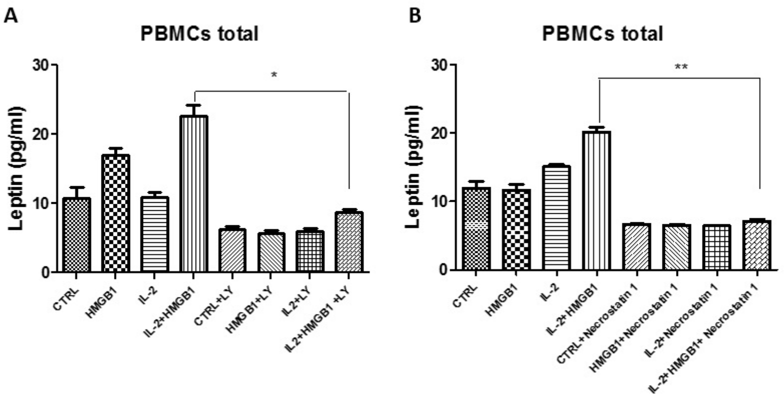

Figure S7. Activation of TLR2 and TLR4 cell signaling increased leptin secretion in isolated PBMCs from healthy donors' buffy coat. LY294002 (30 mM) and Necrostatin-1(100 mM), TLR2 and TLR4 inhibitors, respectively, were added in cells incubated with IL-2 (200 U) for 72 h, just 30 min before HGMB1 treatment. Then, after 48 h of treatment with HMGB1, cells were harvested and supernatants collected to determine leptin concentrations. Inhibition of TLR2 (**A**) and TLR4 (**B**) significantly decreased IL-2/HMB1 mediated leptin secretion. Experiments were performed in triplicate, from. Data are expressed as mean ± SEM and analyzed with Wilcoxon–Mann–Whitney U Test. (* $p < 0.05$, ** $p < 0.005$).

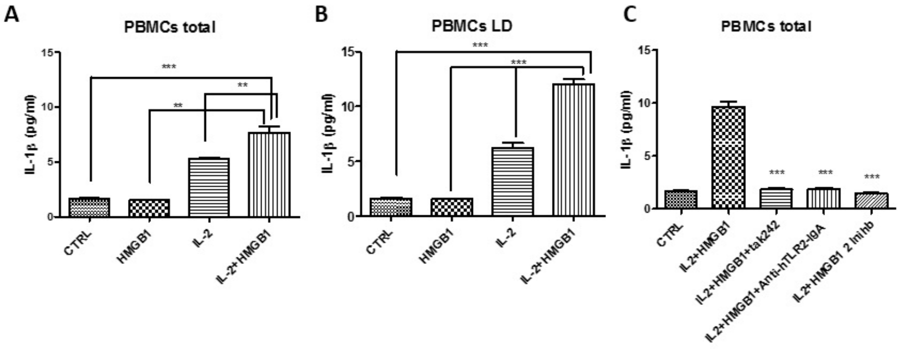

Figure S8. IL-1β secretion was increased in PBMCs after IL-2/HMGB1 co-treatment. Total PBMCs and LD-PBMCs were isolated from human buffy coat and incubated with IL-2 (200 U) for 72 h. Then, IL-2 priming cells were treated with HMGB1 for 48 h; next, cells were harvested, and supernatant was collected for IL-1β determination. IL-1β concentrations were higher in response to IL-2/HMGB1 co-treatment in both total (**A**) and LD-PBMCs (**B**). Furthermore, blocking TLR2 and/or TLR4 signaling with specific inhibitors, a significant decrease in IL-1β concentration was found (**C**). Experiments were performed in triplicate, from healthy donors' buffy coat. Data are expressed as mean ± SEM and analyzed with Wilcoxon–Mann–Whitney U Test. (** $p < 0.005$, *** $p < 0.0005$).

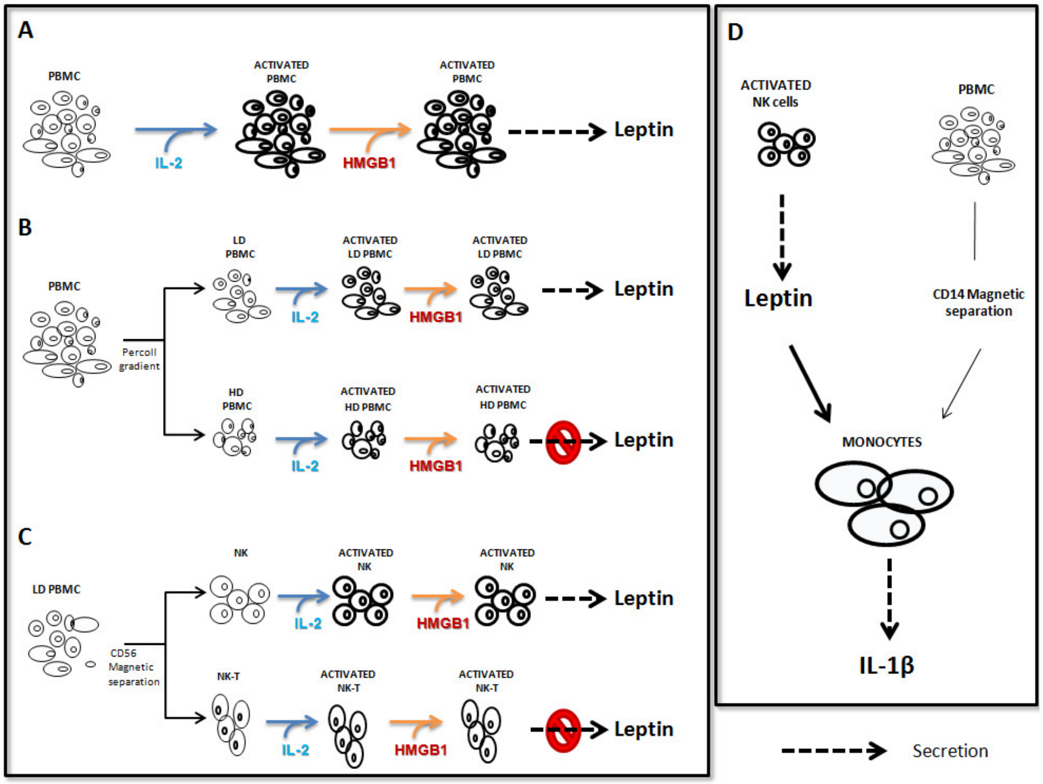

Figure S9. Leptin secretion in response to IL-2 plus HMGB1 proinflammatory stimulus from PBMCs and their subpopulation summary: total PBMCs secrete a moderate amount of leptin after pro-inflammatory stimuli (**A**). Among PBMC subpopulations, low density PBMCs are responsible for leptin secretion after IL-2 plus HMGB1 co-treatment; conversely, high density PBMCs do not increase basal leptin release after treatments (**B**). NK cells secrete the higher amount of leptin after IL-2 plus HMGB1, while NK-T displayed the higher basal leptin release compared to the other PBMC subpopulations (**C**). A slight leptin amount secreted by NK cells after proinflammatory IL-2 plus HMGB1 might induce a significant IL-1β increase from CD14 isolated monocytes.
